# Plant growth-promoting bacteria (PGPB) inoculants enhance the bacterial network connectivity more than non-PGPB in heavy metal-contaminated soil

**DOI:** 10.3389/fpls.2025.1717976

**Published:** 2025-12-17

**Authors:** Zhaoyu Kong, Yong He, Jiaxiang Xue, Zhaohao Chen, Yongqi Gui, Jiahao Wu, Xiaomeng Chen

**Affiliations:** School of Life Science, Key Laboratory of Poyang Lake Environment and Resource Utilization, Ministry of Education, Nanchang University, Nanchang, China

**Keywords:** microbial inoculant, indigenous microbial community, co-occurrence network, rhizosphere colonization, phytoremediation

## Abstract

Optimizing the performance of plant growth-promoting bacteria (PGPB) inoculants in phytoremediation requires a deeper understanding of their interactions with the indigenous soil microbiome. However, current knowledge is particularly limited regarding how PGPB versus non-PGPB inoculants interact with indigenous microbes and establish colonization persistence in the rhizosphere. In this study, we employed amplicon sequencing to compare the impacts of PGPB versus non-PGPB inoculants on the indigenous rhizosphere and bulk soil microbiome of Indian mustard in heavy metal-contaminated soil, and their interactions within the indigenous bacterial networks. Our results showed that both PGPB and non-PGPB inoculants significantly altered the composition and diversity of the rhizosphere microbiome. However, only PGPB inoculants enhanced the complexity and stability of bacterial co-occurrence networks. PGPB inoculants not only maintained rhizosphere persistence but also actively integrated into the network, enhancing associations with indigenous bacterial taxa. Notably, they established enhanced co-occurrence associations with native bacterial taxa characterized as potential PGPB, including *Lysinimonas*, *Sinomonas*, *Nocardioides*, *Actinoalloteichus*, *Amycolatopsis*, *Bradyrhizobium*, *Novosphingobium* etc., and these interactions were predominantly positive. These findings highlight the important role of PGPB versus non-PGPB inoculants in reshaping the rhizosphere microbiome under heavy metal stress, indicating a potential approach for improving phytoremediation efficiency by fostering a more resilient and cooperative soil microbiome.

## Introduction

Plant growth-promoting bacteria (PGPB) refer to free living bacteria in the soil and rhizobacteria that colonize root rhizosphere, exerting beneficial effects on plants. PGPB inoculants are widely used in phytoremediation to improve plant tolerance to heavy metals and enhance soil cleanup efficiency in contaminated environments. Over the past few decades, scientists have developed a much better understanding of how various PGPB alleviate metal-induced phytotoxicity and enhance the biomass of plants grown in heavy metal-contaminated soils (Kong and Glick, 2017). In recent years, significant research attention has been focused on how PGPB inoculation affect the indigenous soil microbial communities. This focus aims to enhance the effectiveness and prevalence of PGPB inoculants, which greatly depend on their ability to colonize and compete with the indigenous microbial community (Thijs et al., 2016). A meta-analysis of 108 studies showed that 86% of cases had measurable impacts of microbial inoculants on native soil microbial communities, and the effects varied from transient shifts to sustained structural alterations (Mawarda et al., 2020). A subsequent meta-analysis of 335 studies further demonstrated a positive impact of microbial inoculants on soil microbial biomass (Li et al., 2024). Even if microbial inoculants fail to colonize, the resulting cell death triggers a nutrient pulse in the soil, which subsequently affects the resident microbial communities (Amor et al., 2020; Mallon et al., 2018). Nevertheless, all the aforementioned research findings are based on comparisons between PGPB and non-inoculated controls, while little attention has been paid to comparisons between PGPB and non-PGPB.

Microbial colonization is a multifaceted and intricate process that involves numerous interactions and mechanisms. Microbial inoculants can perturb this system through direct microbial competition, niche displacement, or indirect modulation of root exudation patterns, thereby altering microbial composition and functional dynamics (Poppeliers et al., 2023). Nutrient competition represents a key mechanism shaping soil microbiome. Plant growth-promoting (PGP) traits including phosphorus solubilization, siderophore production, and ACC deaminase activity allow PGPB to outcompete native soil microbiota for nutrients, which is essential for successful root colonization. However, the interactions between PGPB inoculants and resident soil microbes remain poorly understood, especially in comparison to non-PGPB inoculants. Understanding how PGPB inoculants interact with resident soil bacteria is crucial for identifying synergies or competition between PGPB and indigenous bacteria, thereby promoting plant growth and fitness in heavy metal-contaminated soils. Our previous study showed that PGPB inoculation enhanced the stability and complexity of bacterial co-occurrence networks during phytoremediation of heavy metal-contaminated soil (Kong et al., 2019), which is consistent with the findings reported by Nie et al. (2024). However, these findings are also based on comparisons between PGPB and non-inoculated controls, rather than between PGPB and non-PGPB. In this study, we investigated the impacts of PGPB versus non-PGPB inoculants on indigenous soil bacterial community of Indian mustard (*Brassica juncea* L. Czern.) grown in heavy metal-contaminated soil, and their interactions within indigenous soil bacterial networks. We hypothesized that the interactions between PGPB inoculants and resident soil bacteria would be significantly enhanced as compared to non-PGPB inoculants, thereby improving their persistence in the heavy metal-contaminated soil.

## Materials and methods

### Pot experiments and inoculation

The multiple heavy metal-contaminated soil used in this study was collected from an abandoned farmland (Lat. 28°92′N, Long. 117°48′E) near Dexing Copper Mine, the largest open-cast copper mine in Asia (Teng et al., 2010), located in Jiangxi Province, China. The sampling site has been continuously contaminated with acidic mine drainage for ~60 years with the waste effluents containing Cu, Pb, Cd, Zn, As and Mo from the mine. The collected soil was passed through a 2 mm sieve, homogenized and packed into plastic pots (15 cm diameter, 13 cm height). An 800 g aliquot of soil was packed into each pot and 100 ml Hoagland’s solution was added and allowed to equilibrate for one week.

Indian mustard seeds were surface sterilized by treatment with 75% (v/v) ethanol for 2 min followed by 10 min in 20% (v/v) NaClO (containing 8% available chlorine). After the seeds were thoroughly rinsed with several changes of sterile distilled water, they were planted in each plastic pot. Seven seedlings were planted in each pot and three replicates were conducted for each treatment. To simulate natural lighting conditions, the pots were maintained in a climate chamber at 25 °C in the light for 16 h, and 20°C in the dark for 8 h. The same amount of Hoagland’s solution (80 ml every 4–5 days) was added to each pot to keep the soil wet during the experimental period. After 10 days, the seedlings were inoculated with cell suspensions of *Burkholderia anthina* S6-1 (S6-1), *Pseudomonas putida* UW4 (UW4) or *P. fluorescens* ATCC17400 (negative control, NC), respectively. *B. anthina* strain S6–1 were originally isolated from the sampling site, which possessed both relatively good plant growth promoting activities and multiple heavy metal resistance (Wu et al., 2019). *P. putida* UW4 was originally isolated from the rhizosphere of common reeds on the campus of the University of Waterloo in Canada, based on its ability to utilize ACC as a sole source of nitrogen (Duan et al., 2013; Glick et al., 1995). *P. fluorescens* ATCC17400, obtained from the American Type Culture Collection, was previously reported to have no plant growth-promoting activity (Shah et al., 1998). The plant growth-promoting activities and minimum inhibition concentration of heavy metals of these studied stains are presented in **Table 1**. All of the bacterial inoculants were grown for 2 days at 28 °C with shaking at 150 rpm in nutrient broth. The bacterial cultures were standardized to an optical density of 0.8 at 600 nm (approximately 10^8^ CFU/ml), and 30 ml of the bacterial cell suspension was inoculated into each pot. The inoculum density reached approximately 3.8×10^6^ CFU per gram of soil. Seedlings inoculated with the same amount of sterile distilled water were regarded as the non-inoculated control (CK).

**Table 1:** The plant growth-promoting activities and minimum inhibition concentration (MIC) of heavy metals of used strains in this study.

Strains	ACC deaminase (μm α-KB/mg·h)	Siderophore production (diameter of halo mm)	Phosphate solubilization (diameter of halo mm)	Ammonia Production (mg/l)	MIC of heavy metals (mg/l)			Reference
					Cu^2+^	Pb^2+^	Zn^2+^	
*Burkholderia anthina* S6-1	1.41 ± 0.27	25	11	21.08 ± 1.26	50	1200	3200	Wu et al., 2019
*Pseudomonas putida* UW4	2.35 ± 0.02	nd	12	10.30 ± 1.26	50	300	200	Duan et al., 2013
*Pseudomonas fluorescens* ATCC17400	nd	nd	nd	11.04 ± 0.40	50	300	300	Shah et al., 1998

Results shown as average ± standard deviation. nd: not detective.

### Soil sampling and analysis

The inoculated soils were collected at 40 days after inoculation. Individual plants were gently uprooted and soil adhering the root system was shaken off. The soils tightly adhering to the roots were defined as rhizosphere soil. At the same time, the bulk soil (defined as the root-free soil) was collected from each pot and served as a reference for the rhizosphere soil samples. One subset of rhizosphere/bulk soil samples was air-dried and analyzed for soil properties and heavy metal contents. The other subset of rhizosphere/bulk soil samples was stored at -80 °C for DNA extraction. The soil pH value was measured in 1:3 (W/V) fresh soil: distilled water using a pH meter (SG2, Mettler Toledo Instruments Co. Ltd., Shanghai, China). According to preliminary assessment (Wu et al., 2019), the sampling site were primarily contaminated with copper (Cu), zinc (Zn), and lead (Pb). Soil total Cu, Pb and Zn were extracted with concentrated sulfuric acid (H_2_SO_4_), and the metal concentrations in the extracts were determined with an inductively coupled plasma-optical emission spectrometer (ICP-OES) (Optima 3000, Pekin-Elmer, Wellesley, USA). Soil moisture (SM) was measured by oven-drying for 24 hours at 105 °C. The soil nutrient contents, including total nitrogen (TN), available nitrogen (AN), total phosphorus (TP), available phosphorus (AP), total potassium (TK) and available potassium (AK) were measured using standard soil testing procedures (Bao, 2000). Ash-free dry mass (AFDM) was measured by incineration at 550°C for 4 hours. Total organic carbon (TOC) content was determined using the K_2_Cr_2_O_7_/H_2_SO_4_ oxidation procedure. The soil from the sampling site is classified as Ferrosols according to the Chinese Soil Taxonomy (2001). The soil texture was determined to be sandy loam based on the measured sand-silt-clay ratios (67.20% sand, 26.03% silt, and 6.77% clay). The soil physical and chemical parameters are presented in **Supplementary Table S1**.

### DNA preparation and MiSeq sequencing

Total genomic DNA was extracted from each of the rhizosphere and bulk soil samples using a Power Soil DNA Isolation Kit (MOBIO Laboratories, Inc., Carlsbad, CA, USA) following the manufacturer’s instructions. The V3-V4 hypervariable region of the 16S rRNA gene was amplified using the primers 338F (5’-GTGCCAGCMGCCGCGGTAA-3’) and 806R (5’-GGACTACHVGGGTWTCTAAT-3’) as previously described (Zhang et al., 2018). PCR amplification was conducted in triplicate and the PCR products from each soil sample were gel-purified and sequenced on the Illumina MiSeq platform by Personal Biotechnology, Co., Ltd. (Shanghai, China). Raw sequence data were quality filtered and analyzed using Trimmomatic (Version 0.36). Primers, contaminating adaptor and unpaired reads were discarded. Quality sequences were aligned in accordance with SILVA alignment and clustered into operational taxonomic units (OTUs) using UPARSE version 9.2 (Edgar, 2013). Taxonomical assignments of the OTUs with 97% sequence similarity were performed using the Mothur program in accordance with SILVA (Silva.nr_v132) at a confidence level of 80%. Data sets of the 16S rRNA gene sequences were deposited in the National Center for Biotechnology Information (NCBI) Sequence Read Archive under accession ID PRJNA1295673.

### Statistical and network analysis

Soil properties were analyzed using principal component analysis (PCA) by Canoco 5.0 (Ter Braak and Šmilauer, 2012), based on the correlation matrix from the log-transformed soil data set. The rarefaction curve and α-diversity indices, including the Chao, Shannon, and Simpson indices, were analyzed at the OTU level using the Mothur program (Schloss et al., 2009). Principle coordinates analysis (PCoA) based on Bray-Curtis and weighted UniFrac distances was performed to evaluate the differences in microbial communities among samples, using the vegan package in R software. The significance of PCoA clustering patterns was assessed using an analysis of similarity (ANOSIM) (Clarke, 1993). The statistical significance of the differences between the means of samples was determined by one-way ANOVA, also using R software.

To investigate the co-occurrence patterns of soil bacterial taxa across various inoculation treatments, network analysis was conducted without differentiating between rhizosphere and bulk soil. A correlation matrix was created by calculating all pairwise Spearman’s rank correlations among bacterial operational taxonomic units (OTUs). OTUs with a relative abundance greater than 0.05% were chosen for the analysis. P-values were adjusted using the Benjamini and Hochberg false discovery rate (FDR) test (Benjamini et al., 2006), and the adjusted P-values had a 0.05 threshold. The topological properties of the networks were computed using the igraph package (Csardi, 2006), and the networks were visualized through the interactive platform Gephi (Bastian et al., 2009). Each node and edge in the network represented an OTU and a strong, significant correlation between OTUs, respectively. Additionally, 10,000 Erdős-Rényi random networks of equal size were generated for comparison to assess the topology of the real networks (Erdős and Rényi, 2012).

## Results

### PGPB inoculant effects on the physicochemical properties of bulk and rhizospheric soils

Variations of overall soil properties among samples were assessed by principal components analysis (PCA) based on 11 physicochemical variables. PC1 and PC2 captured 76.35% and 13.38% of the total variance of soil physicochemical variables, respectively (**Supplementary Figure S1**). As illustrated by the PCA plot, rhizospheric soils from all inoculant treatments were distinctly separated from the corresponding bulk soil samples, while the CK treatment did not. Moreover, rhizospheric and bulk soil samples from the S6–1 treatment formed a distinct cluster separate from other treatments, corresponding to significant shifts in soil physicochemical properties under different inoculant treatments (**Supplementary Table S2**).

### PGPB inoculant effects on the alpha diversity of bacterial community

A data set of 1,075,557 quality sequences was obtained from all bulk and rhizosphere soil samples. A total of 1460 OTUs, defined at 97% sequence similarity, were annotated to 26 phyla, 64 classes, 154 orders, and 264 families. The number of OTUs per sample ranged from 799 to 948 for bulk soil samples, and from 710 to 925 for rhizosphere soil samples (**Supplementary Table S3**). The Good’s estimator of coverage (how well a sample represents the population) ranged from 99.47% to 99.84%, demonstrating that most of the bacterial taxa in soil samples were detected. The rarefaction curves at the OTU level reached plateau, suggesting high recovery of microbial diversity by obtained sequences (**Supplementary Figure S2**). Across all the samples, Actinobacteria (34.86%) and Proteobacteria (30.42%) represented the most dominant phyla. Some less abundant phyla were also detected in all the samples, including Chloroflexi (9.06%), Patescibacteria (6.93%), Acidobacteria (5.93%), Firmicutes (4.42%), Bacteroidetes (2.65%), and Planctomycetes (2.03%). The most dominant classes included Actinobacteria (24.73%), Gammaproteobacteria (17.27%), and Alphaproteobacteria (12.34%). At the genus level, the most dominant phylotypes were *Dyella* (7.04%), followed by *Saccharimonadales_ge* (6.32%), *Frankia* (5.30%), and *Sphingomonas* (4.51%).

**Figure 1:**
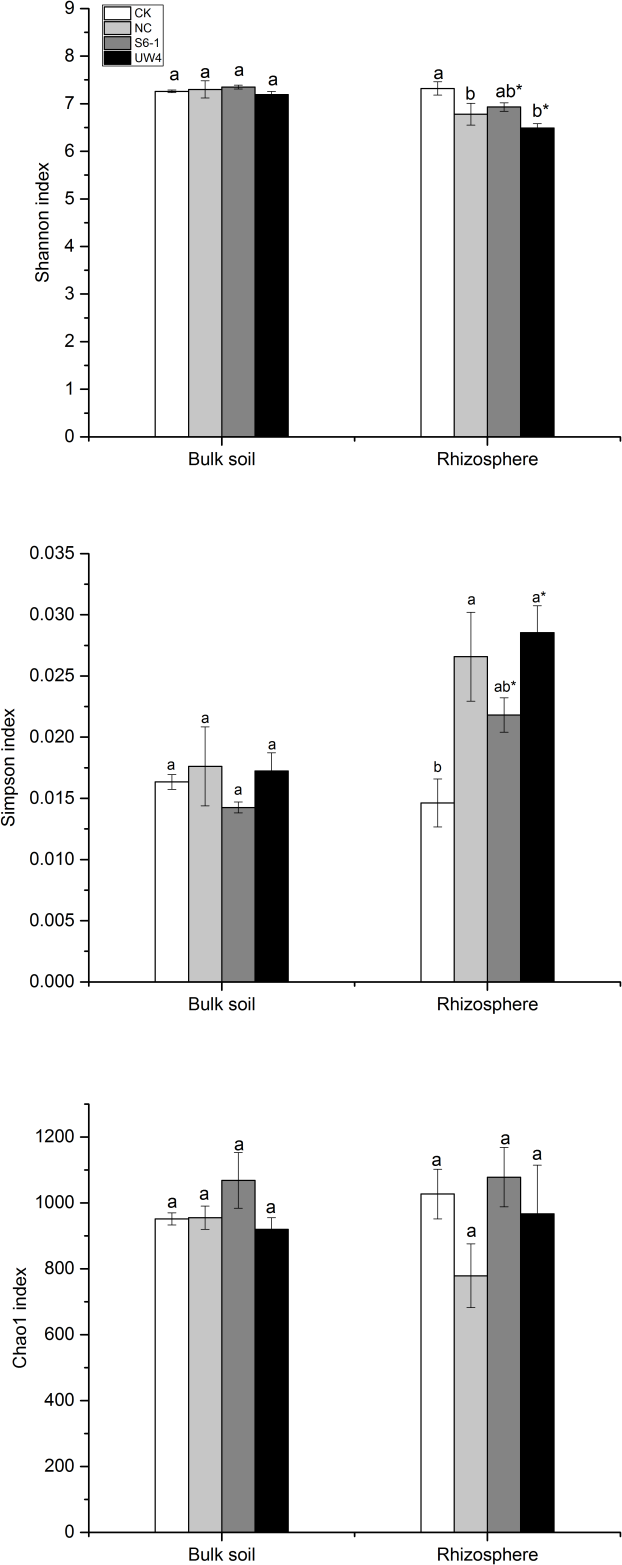
The estimators of Shannon, Simpson and Chao1 diversity of bulk (B) and rhizosphere (R) soil bacterial community under different PGPB inoculation treatments. Different letters indicate statistically significant differences (p<0.05) among different inoculation treatments by Duncan test. *Statistically significant difference (p<0.05) for the B and R soil samples under the same inoculation conditions by T-test. S6-1, inoculation with the PGPB strain *B anthina* S6-1; UW4, inoculation with the PGPB strain *Pseudomonas putida* UW4; NC, inoculation with the non-PGPB strain *P. fluorescens* ATCC17400; CK, non-inoculated control.

No significant difference in alpha-diversity was observed among different treatments for bulk soil samples (ANOVA, p>0.05; **Figure 1**). Nevertheless, for the rhizosphere bacterial community, a reduced alpha-diversity, as evidenced by the Shannon and Simpson indices, was observed across all inoculated treatments when compared to the CK treatment. Additionally, the bacterial community diversity in the PGPB-inoculated rhizospheric soil was significantly lower compared to that in the bulk soil.

### PGPB inoculant effects on the beta diversity of bacterial community

**Figure 2:**
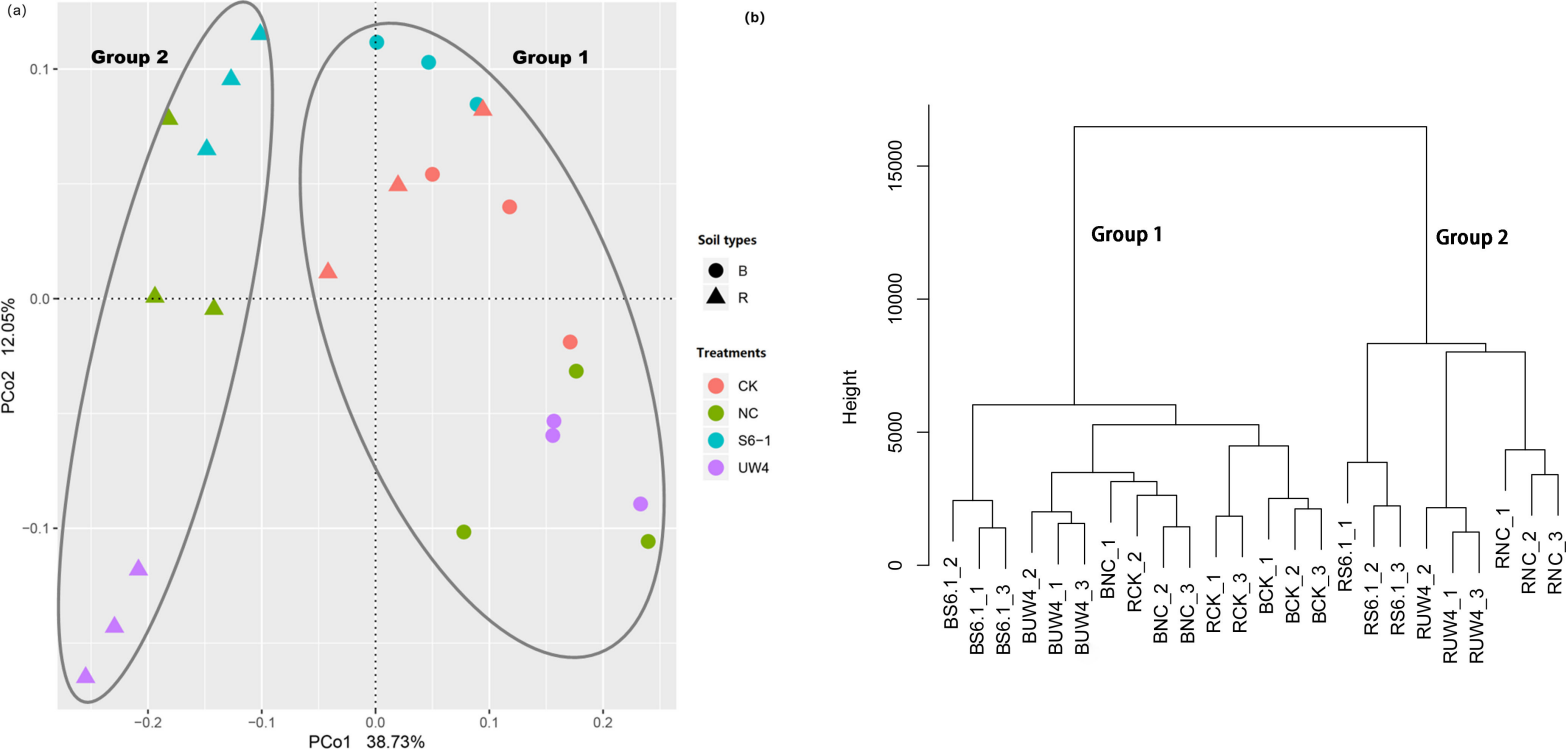
**(a)** Principle coordinates analysis (PCoA) and **(b)** cluster analysis derived from Bray-Curtis distance matrix, based on the relative abundances of the bacterial OTUs. The values of axes 1 and 2 are the percentages that can be explained by the corresponding axis. B, bulk soil; R, rhizosphere. S6-1, inoculation with the PGPB strain *B. anthina* S6-1; UW4, inoculation with the PGPB strain *Pseudomonas putida* UW4; NC, inoculation with the non-PGPB strain *P. fluorescens* ATCC17400; CK, non-inoculated control.

Both PCoA and cluster analyses revealed that, regardless of the presence of PGPB, all inoculated rhizosphere samples were distinctly separated from their corresponding bulk soil samples (**Figure 2ab**). whereas the CK treatment exhibited no such separation. The differences in bacterial community composition between rhizosphere and bulk soil were tested using ANOSIM (R_ANOSIM_=0.60; p < 0.001). The ANOSIM test also revealed that the bacterial community structure of all bulk soil samples and rhizosphere soil of the CK treatment (Group 1) and all inoculated rhizosphere soil samples (Group 2) was significantly different (R_ANOSIM_=0.77; p < 0.001). This indicated that apart from the non-inoculated treatment, both PGPB and non-PGPB inoculants significantly altered the structure of the rhizosphere microbiome. Moreover, the UW4 treatment was separated from the S6–1 and NC treatments. The first two axes (PC1 and PC2) explained 38.73% and 12.05%, respectively, of the total variance in the bacterial community.

**Figure 3:**
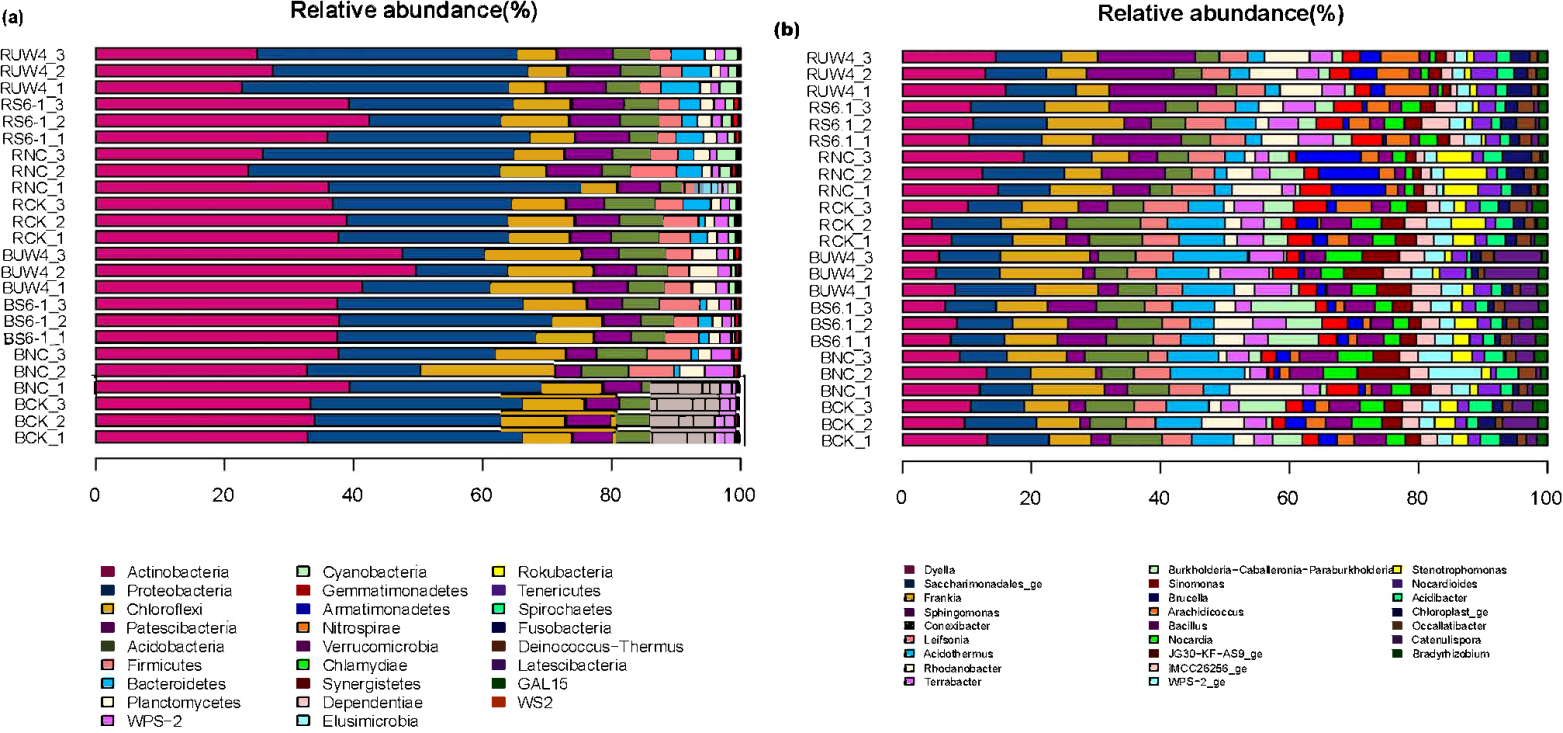
The changes in bacterial community composition under various inoculation treatments. **(a)** At the phylum level; **(b)** At the genus level (>1%). B, bulk soil; R, rhizosphere. S6-1, inoculation with the PGPB strain *B. anthina* S6-1; UW4, inoculation with the PGPB strain *Pseudomonas putida* UW4; NC, inoculation with the non-PGPB strain *P. fluorescens* ATCC17400; CK, non-inoculated control.

The changes in the rhizosphere bacterial community composition under various inoculation treatments were further analyzed (**Figures 3a, b**). Both PGPB and non-PGPB inoculants increased the relative abundance of Bacteroidetes and Patescibacteria, and PGPB had a more obvious effect (**Figure 3a**). At the genus level, both PGPB and non-PGPB treatments enhanced the relative abundance of *Dyella*, *Sphingomonas*, *Leifsonia*, *Rhodanobacter*, *Nocardioides*, *Ensifer*, and *Singulisphaera*. Among them, *Sphingomonas*, *Nocardioides*, *Ensifer* and *Singulisphaera* were significantly improved under PGPB inoculation (**Figure 3b**). In contrast, PGPB inoculation reduced the relative abundance of *Stenotrophomonas*, *Acinetobacter*, *Microbacterium*, *Tumebacillus*, *Novosphingobium* compared with CK or NC treatments.

### PGPB inoculant effects on the co-occurrence patterns of soil bacterial community

**Figure 4:**
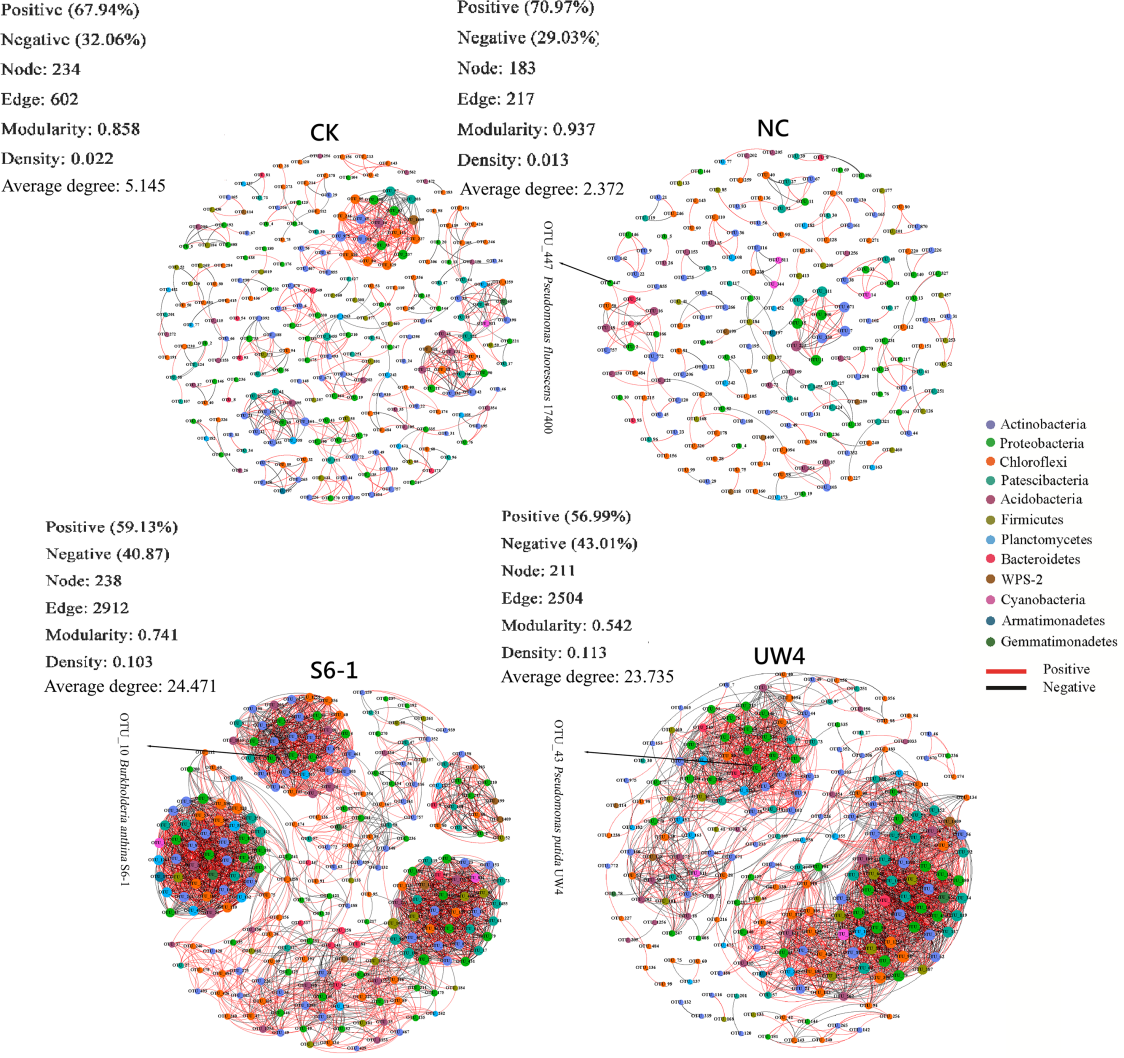
Network of co-occurring bacterial OTUs under different inoculation treatments based on correlation analysis. A connection indicates a statistically significant (p < 0.05) positive (red, Spearman’s ρ> 0.6) or a negative correlation (black, Spearman’s ρ< −0.6). The size of each node is proportional to the number of connections; the nodes of the same color were affiliated with the same phylum; and the thickness of each connection between two nodes is proportional to the value of Spearman’s correlation coefficients of ρ > 0.6 or < -0.6. The OTU indicated by the arrow perfectly matches the 16S rRNA gene sequence of the inoculated strains. S6-1, inoculation with the PGPB strain *B. anthina* S6-1; UW4, inoculation with the PGPB strain *Pseudomonas putida* UW4; NC, inoculation with the non-PGPB strain *P. fluorescens* ATCC17400; CK, non-inoculated control.

The co-occurrence networks of soil bacterial community under different bacterial inoculation treatments were explored based on strong and significant correlations (Spearman’s correlation, r > 0.6 or <-0.6, and p < 0.05) (**Figure 4**; **Supplementary Table S4**). The modularity values of all treatments were higher than 0.4, suggesting that each network has a modular structure. Moreover, the network structural properties (modularity, average clustering coefficient and average path length) from all treatments were greater than those found in Erdös-Réyni random networks of equal size, indicating that the networks have “small world” properties (**Supplementary Table S4**). Multiple network topological properties consistently showed that bacterial co-occurrence patterns of PGPB-inoculated treatments differed profoundly from the networks for CK or NC treatments. The networks for S6–1 and UW4 contained significantly more connections (2912 and 2504 edges, respectively) between nodes when compared with CK (602 edges) or NC (217 edges) networks. Moreover, PGPB-inoculated networks had significantly higher average degree and greater graph density (**Figure 4**; **Supplementary Table S4**).

**Figure 5:**
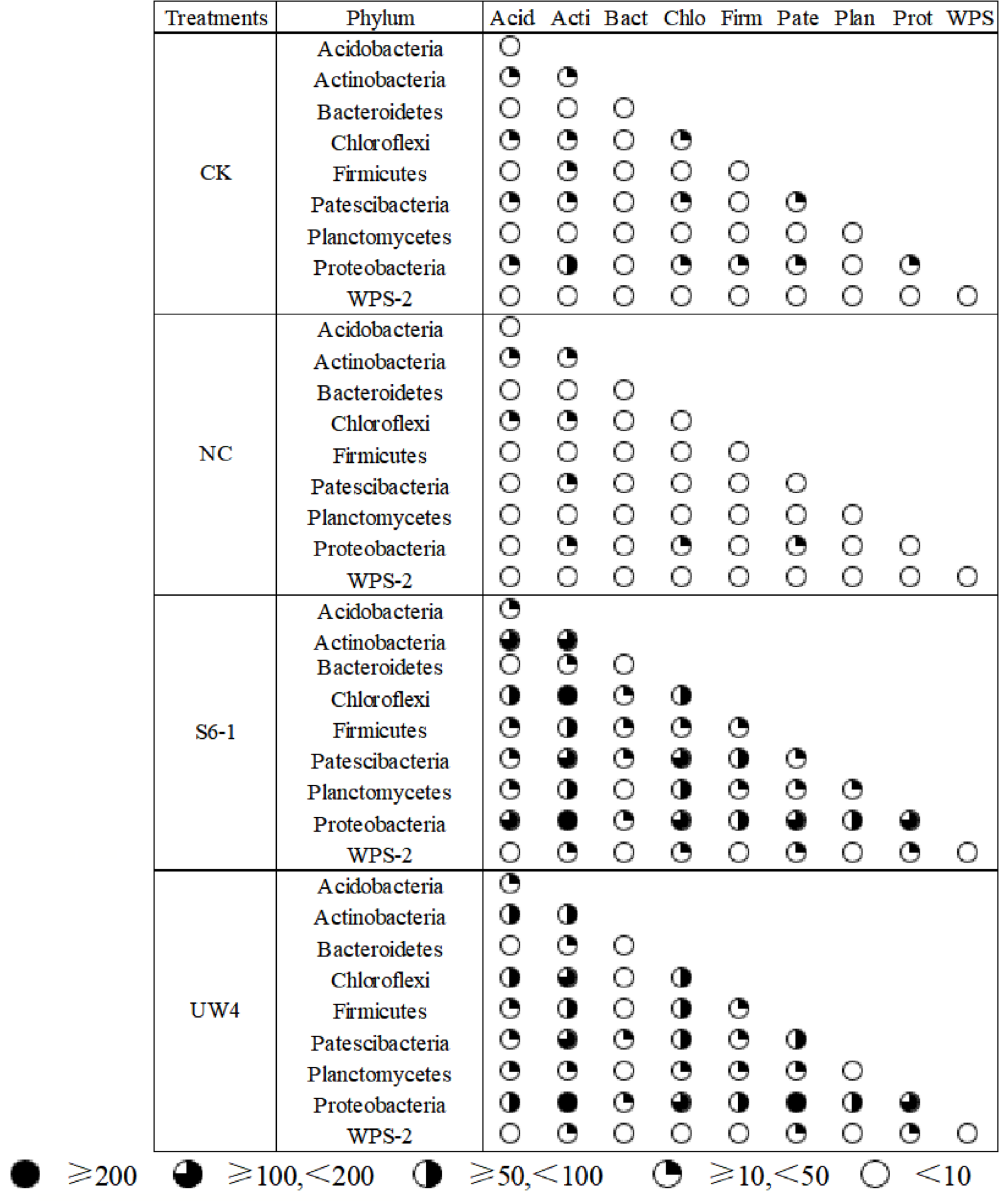
Co-occurrence association (targeted edges) inter-phyla and intra-phylum under different inoculation treatments. The size of the black sector area is proportional to the number of inter-phylum or intra-phylum connections. S6-1, inoculation with the PGPB strain *B. anthina* S6-1; UW4, inoculation with the PGPB strain *Pseudomonas putida* UW4; NC, inoculation with the non-PGPB strain *P. fluorescens* ATCC17400; CK, non-inoculated control.

Most nodes (97.27%-98.72%) in the networks for all four treatments were assigned to 9 bacterial phyla, including Acidobacteria, Actinobacteria, Bacteroidetes, Chloroflexi, Firmicutes, Patescibacteria, Planctomycetes, Proteobacteria and WPS-2. PGPB inoculation significantly enhanced the associations within and among these bacterial phyla, as compared to the CK and NC treatments (**Figure 5**). In addition, the networks for S6–1 and UW4 had more negative correlations (40.87% and 43.01%, respectively) than CK (32.06%) or NC (29.03%) networks. These results revealed that the bacterial community of S6–1 and UW4 had more complex and compact associations than CK and NC.

### The survival and persistence of PGPB inoculants in the rhizosphere

**Figure 6:**
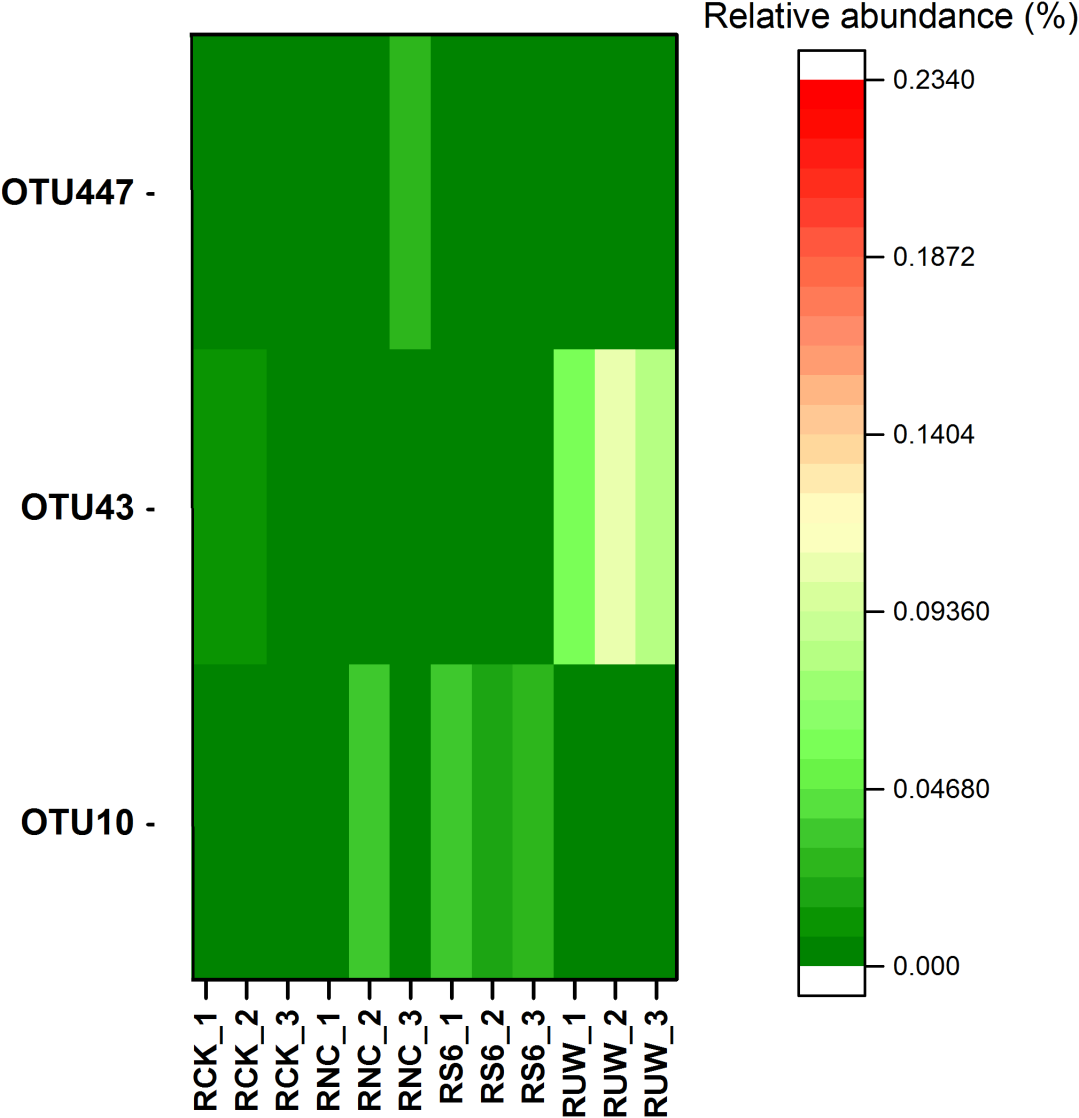
The relative abundance of OTU 447, OTU43, OTU10 in the rhizospheric soil samples under different inoculation treatments. The OTUs 447, 43 and 10 were defined as *P. fluorescens* ATCC17400, *P. putida* UW4 and *B. anthina* S6-1, respectively.

To track the survival of the inoculated strains, we further identify the OTUs that perfectly matched the 16S rRNA gene sequence of the inoculated strains. The results showed that OTU10, OTU43, and OTU447 could perfectly match the 16S rRNA gene sequence of *B. anthina* S6-1 (99.56% similarity), *P. putida* UW4 (99.56% similarity) and *P. fluorescens* ATCC17400 (99.78% similarity), respectively. Furthermore, the inoculation of *P. putida* UW4 and *B. anthina* S6–1 significantly elevated the relative abundance of OTU43 and OTU10, respectively, within the rhizosphere of the UW4 and S6–1 treated plants (**Figure 6**). These results suggested that we likely defined the actual OTUs that were previously inoculated. However, little increase was observed in the relative abundance of OTU447 in the rhizosphere under the treatment of inoculation with non-PGPB strain *P. fluorescens* ATCC17400.

### Characterization of recruited bacteria by PGPB inoculation

**Figure 7:**
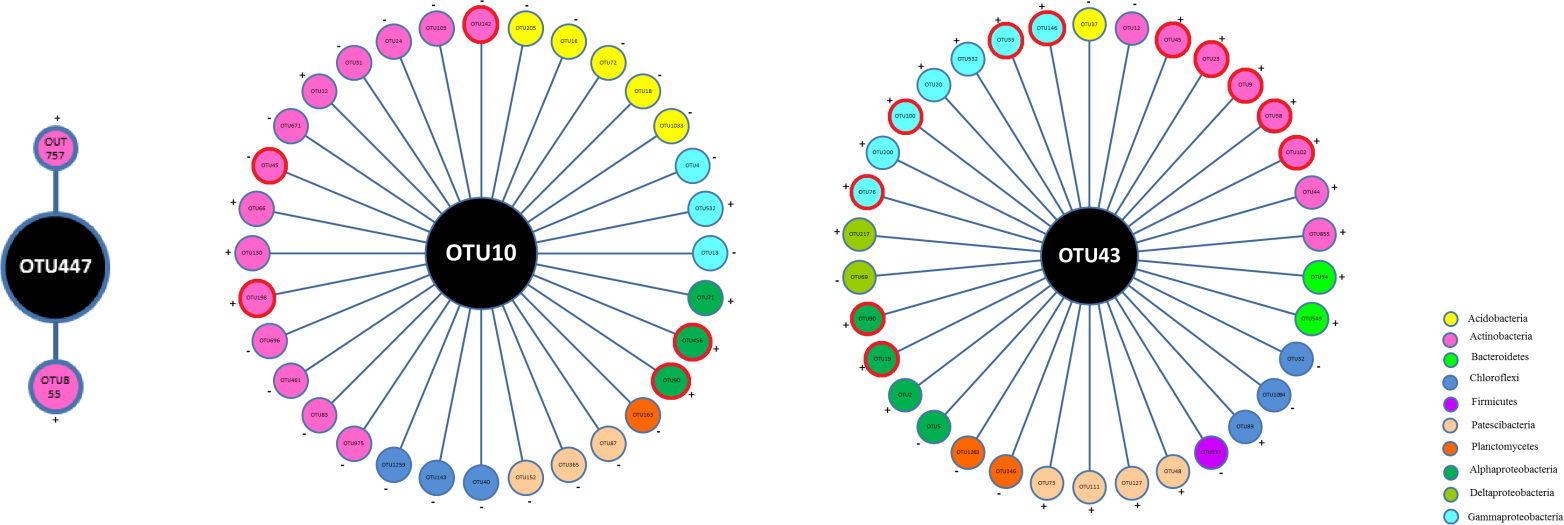
Co-occurrence associations between the defined OTUs and other indigenous bacterial taxa. The OTUs 447, 10 and 43 were defined as *P. fluorescens* ATCC17400, *B. anthina* S6–1 and *P. putida* UW4, respectively. The nodes of the same color were affiliated with the same phylum. The ‘+’ and ‘-’ outside the OTU circles represent positive and negative correlations, respectively. The OTU enclosed in a red bold circle represents a potential PGPB according to the PGPB database constructed by Li et al. (2023).

To further characterize the interactions between bacterial inoculants and indigenous bacteria, we particularly analyze the co-occurrence associations between the defined OTUs and other indigenous bacterial taxa (**Figure 7**; **Supplementary Table S5**). The association number between OTU10 and other taxa was 32 in the S6–1 network, with the majority of taxa (75.0%) exhibiting negative interactions with OTU10. The associations between OTU43 and other taxa numbered 34 within the UW4 network, with the majority of these taxa (73.5%) exhibiting positive interactions with OTU43. However, the number of associated links between OTU447 and other taxa were only 2 in the NC network. In the network for CK treatment, there was only a slight association (7) between OTU43 and other bacterial OTUs, only 2 links between OTU447 and other OTUs, and no links could be accounted between OTU10 and other OTUs.

Among the co-occurrence associations for OTU10 in the S6–1 network, a total of 14 links (43.75%) could be accounted between OTU10 and the Actinobacterial taxa. A total of 5 links (15.63%) were observed between OTU10 and the Acidobacterial OTUs. The other associations could be accounted between OTU10 and the OTUs belonging to Chloroflexi (3 links), Patescibacteria (3 links), Planctomycetes (1 links), Gammaproteobacteria (3 links) and Alphaproteobacteria (3 links). Among the associations for OTU43 in the network UW4, a total of 8 links (23.53%) could be accounted between OTU43 and the Actinobacterial taxa, and 7 links (20.59%) could be observed between OTU43 and the OTUs belonging to Gammaproteobacteria. Some associations were observed between OTU43 and the OTUs belonging to Alphaproteobacteria (4 links), Patescibacteria (4 links), Chloroflexi (3 links), Bacteroidetes (2 links), Deltaproteobacteria (2 links), Planctomycetes (2 links), Acidobacteria (1 link), and Firmicutes (1 link). Further taxonomic annotation of the associated OTUs was provided in Supplementary Materials **Supplementary Table S5**.

According to the PGPB database constructed by Li et al. (2023), 11 bacterial OTUs associated with OTU43 were characterized as potential PGPB, including *Lysinimonas*, *Sinomonas*, *Nocardioides*, *Actinoalloteichus*, *Amycolatopsis*, *Bradyrhizobium*, *Novosphingobium* etc. Notably, all these potential PGPB exhibited positive interactions with OTU43. Additionally, five potential PGPB OTUs were found to be associated with OTU10. While, no PGPB genera were associated with OTU447.

## Discussion

The alterations in the diversity and composition of the rhizosphere microbial community, resulting from inoculation with PGPB, are considered closely related to their efficacy in promoting plant growth (Kong and Liu, 2022). These alterations even provide a more comprehensive explanation for PGPB beneficial effects on host plants, compared to the commonly recognized growth-promoting traits of PGPB alone (Hu et al., 2021). In this study, whether PGPB or non-PGPB, all inoculated rhizosphere samples showed significant difference from their corresponding bulk soils in terms of physicochemical properties and microbiome characteristics. While the CK treatment showed no significant difference between rhizosphere and bulk soil samples. In a bioaugmentation process, to ensure the efficient achievement of positive effects, a large amount of inoculant is always introduced into the soil environment. An inoculation concentration of up to 10^6–^10^8^ CFU/seedling or 10^9–^10^15^ CFU/g soil is often used (Kong and Liu, 2022), which is close to or even exceeds the total number of soil microorganisms (Bloem et al., 1995). The introduction of high-density inoculants can profoundly alter rhizosphere ecology. Even if microbial colonization fails, the resulting cell death triggers a nutrient pulse into the soil matrix. The transient nutrient flux, composed of cellular constituents and metabolic byproducts, serves as a deterministic force reshaping the structure and function of the indigenous rhizosphere microbiome. In this regard, non-PGPB inoculants can also induce structural changes in rhizosphere microbiome through nutrient-mediated pathways. Nevertheless, PGPB inoculants significantly enhanced the relative abundance of potential beneficial genera, such as *Sphingomonas*, *Nocardioides*, *Ensifer* and *Singulisphaera*, which are increasingly recognized for their potential to enhance soil fertility and plant health (Asaf et al., 2020; Qiao et al., 2023; Wang X. et al., 2023; Zhao et al., 2022). Moreover, PGPB inoculation decreased the abundance of genera, such as *Stenotrophomonas*, *Acinetobacter*, *Tumebacillus*, *Novosphingobium*, and *Microbacterium*-some of which are associated with plant disease or reduced plant health. For example, inoculation with synthetic or selected PGPB communities in crops like water yam and *Atractylodes lancea* led to a significant reduction in *Stenotrophomonas* and *Novosphingobium* in the root and rhizosphere microbiomes, suggesting a suppression of potentially harmful or less beneficial bacteria (Liswadiratanakul et al., 2023; Wang et al., 2022).

Microbial inoculants can also serve as a deterministic filtering factor that reshape microbial interactions across trophic levels (Liu and Salles, 2024). To further elucidate the differential impacts of PGPB versus non-PGPB on the soil microbiome, we investigated their persistence and interactions within indigenous soil microbial networks. Our results showed that bacterial co-occurrence patterns of PGPB-inoculated treatments differed profoundly from the networks for non-PGPB or control treatments. Specifically, both PGPB inoculants significantly enhance the complexity and stability of bacterial co-occurrence networks, whereas non-PGPB inoculants do not exhibit such an effect. Higher microbial network complexity (e.g., greater graph density, higher average degree) is strongly associated with enhanced ecosystem multifunctionality, including improved nutrient cycling (C, N, P, S), plant growth, and stress resilience. For example, metagenomic analyses show that more complex networks harbor higher relative abundances of genes for C, N, P, and S cycling, directly linking network structure to functional potential (Guo Y. et al., 2022). Complex networks support greater functional redundancy and diversity, which buffer against environmental disturbances and maintain multiple ecosystem functions (Chen et al., 2022; Jiao et al., 2021; Wagg et al., 2019).

Moreover, the networks for both PGPB inoculated treatments had more negative correlations than non-PGPB or CK networks. Since even genera with low abundance can maintain network stability and function (Guo B. et al., 2022), negative correlations in PGPB-inoculated networks are more likely due to intensified microbial interactions and niche competition, not simply the loss or reduction of certain genera. In fact, the importance of negative microbial interactions in community assembly has been experimentally confirmed, with 39% of the dominant bacterial taxa experiencing competitive interactions during soil recolonization. A community with a large proportion of positive links is considered unstable because it may respond in unison to environmental fluctuations, resulting in positive feedback and co-oscillation (Coyte et al., 2015). In contrast, negative links can stabilize co-oscillation within a community and promote network stability (Coyte et al., 2015; De Vries et al., 2018). These findings indicated that PGPB inoculants enhanced the resilience of soil bacterial community to external disturbances, ensuring the survival of microorganisms (Yuan et al., 2021), whereas non-PGPB inoculants did not. Further characterization of interactions between PGPB inoculants and indigenous bacteria has validated our hypothesis. It revealed that PGPB inoculants not only persisted effectively in the heavy metal-contaminated soil but also actively integrated into the co-occurrence network, enhancing associations with indigenous bacterial taxa. In contrast, non-PGPB inoculants exhibited poor persistence and did not achieve comparable network integration. It is known that the ability to utilize nutrients from root exudates is crucial for rhizobacteria to occupy niches in the rhizosphere (Liu et al., 2024). Therefore, PGPB traits that enhance competitiveness in nutrient acquisition-such as phosphorus solubilization, siderophore production, and ACC deaminase activity-result in higher rhizosphere colonization efficiency. For example, ACC deaminase-producing strains S6–1 and UW4 can degrade ACC as a nitrogen source, providing them with a significant advantage in rhizosphere colonization (Liu et al., 2019), whereas non-PGPB strain 17400 lacks this competitive advantage.

Furthermore, PGPB inoculants have the potential to alter specific functional subgroups within the rhizosphere microbiome. For instance, the inoculation with PGPB strain *Neorhizobium huautlense* T1–17 significantly increased the proportion of IAA-producing bacteria in the rhizosphere of Chinese cabbages and radishes (Wang et al., 2016). The inoculation of phosphate-solubilizing PGPB strains selectively enriched dominant microbial taxa possessing PGP traits and keystone taxa associated with Cd mobilization during soil Cd phytoremediation (He et al., 2022). Similarly, enhanced co-occurrence associations were also observed between PGPB inoculants and bacterial taxa characterized as potential PGPB according to the PGPB database constructed by Li et al. (2024). Moreover, these interactions were predominantly positive for both PGPB strains S6–1 and UW4. These findings indicated the intricate interplay between PGPB inoculants and the indigenous rhizosphere microbiome (Kong et al., 2025). Also, it reminds us that although the colonization of PGPB may result in competitive pressure on the indigenous soil microbiome, the cooperation between native and inoculated PGPB strains cannot be ignored. In fact, an increasing number of cooperative behaviors are being discovered in natural microbial communities, such as metabolic cross-feeding, which significantly affect the fitness of interacting partners (Ma et al., 2024; Semenec et al., 2023; Wang Y. et al., 2023). Moreover, PGPB inoculants can also indirectly interact with native rhizobacteria by modulating the quantity and chemical composition of plant root exudates (Kong et al., 2025). For example, PGPB inoculation can significantly influence the activities and abundances of denitrifying rhizosphere microbiome by modulating root exudates (Florio et al., 2019). Therefore, PGPB inoculants may affect the composition of the rhizosphere microbiome through direct “microbe-microbe” interactions or indirect “microbe-plant-microbe” interactions. Both pathways may alter the functional activity of the rhizosphere microbiome, ultimately facilitating plant growth and fitness during phytoremediation. In this regard, the three-way interactions among PGPB inoculants, the indigenous rhizosphere microbiome, and plant roots need to be studied integratively to understand the plant growth-promoting mechanisms. Although this study revealed distinct microbial co-occurrence patterns in response to PGPB inoculation, our analysis did not identify a significant correlation between network topologies and heavy metals concentrations in the soil. Future research should aim to fill this gap by linking network properties with plant and soil parameters (such as biomass, metal uptake, and enzyme activities). This will help ascertain whether a “more connected” or “more complex” microbiome can result in a more effective and resilient phytoremediation system. A comprehensive understanding of PGPB-mediated rhizosphere microbiome modulation will facilitate the development of targeted rhizosphere engineering strategies for enhanced heavy metal phytoremediation. However, the non-PGPB treatment in our study is represented by a single bacterial strain. The differences observed between the non-PGPB and PGPB treatments may be due to the absence of PGP traits, but they could also result from other inherent genetic, metabolic, or ecological characteristics specific to *P. fluorescens* ATCC17400. To legitimately claim that the effects are due to “PGPB versus non-PGPB,” future research will require the inclusion of multiple, phylogenetically diverse representatives within each category.

## Conclusions

Bacterial inoculants, whether they are PGPB or non-PGPB strains, significantly affected the structure of the rhizosphere microbiome of Indian mustard in heavy metal-contaminated soil. However, only PGPB inoculants were associated with the complexity and stability of bacterial co-occurrence networks. Specifically, PGPB inoculants not only persisted effectively in the rhizosphere but also actively integrated into the co-occurrence network, enhancing associations with indigenous bacterial taxa. Notably, there were enhanced positive associations between PGPB inoculants and native bacterial taxa identified as potential PGPB. These findings highlight the important role of PGPB versus non-PGPB inoculants in reshaping rhizosphere microbiome under heavy metal stress, offering a promising strategy to improve phytoremediation efficiency by fostering a more resilient and cooperative rhizosphere microbiome. However, these co-occurrence patterns are statistically determined based on the associations among the relative abundances of various bacterial OTUs, which can only indicate potential positive, negative or neutral interactions. To robustly investigate microbial networks following PGPB inoculation, future research should integrate metagenomics, metabolomics, and proteomics with network analysis. This approach can link community shifts to functional changes, offering mechanistic insights beyond mere statistical associations.

## Data Availability

The 16S rRNA gene sequence data are accessible in the National Center for Biotechnology Information (NCBI) Sequence Read Archive (SRA), under accession ID PRJNA1295673.
