# Supplementary material for: Plant growth-promoting bacteria (PGPB) inoculants enhance the bacterial network connectivity more than non-PGPB in heavy metal-contaminated soil
Source: Front Plant Sci. 2025 Dec 17;16:1717976. doi: 10.3389/fpls.2025.1717976 (PMC12753923; doi:10.3389/fpls.2025.1717976)
Supplement: Supplementary file 1 [file Supplementaryfile1.docx]

Table S1 The physical and chemical parameters of the soil used in the pot experiment

| pH | 4.56±0.05 |
| --- | --- |
| AFDM (%) | 3.48±0.02 |
| TOC (g/kg) | 18.11±0.95 |
| TN (g/kg) | 0.75±0.05 |
| TP (mg/kg) | 53.46±6.54 |
| Cu (mg/kg) | 92.81±1.10 |
| Zn (mg/kg) | 67.95±0.98 |
| Pb (mg/kg) | 21.97±0.36 |

Results shown as average ± standard deviation of three replicates (n=3). AFDM: ash-free-dry-mass; TN: total nitrogen; TP: total phosphorus; TOC: total organic carbon.

Table S2 The physichemical properties of bulk (B) and rhizosphere (R) soil samples under different PGPB inoculation treatments (mean ± standard variation)

| Habitat | Treatments | SM (%) | pH | Cu (mg/kg) | Pb (mg/kg) | Zn (mg/kg) | TN (g/kg) | TP (g/kg) | TK (g/kg) | AN (mg/kg) | AP (mg/kg) | AK (mg/kg) |
| --- | --- | --- | --- | --- | --- | --- | --- | --- | --- | --- | --- | --- |
| B | CK | 16.81±1.53 a | 4.72±0.02a | 53.89±2.07a | 44.13±0.83 a | 83.76±3.54 a | 0.78±0.03 a | 0.58±0.00 a | 19.62±0.22 a | 25.94±0.10 a | 254.43±21.27 a | 84.95±7.40 bc |
|  | NC | 14.45±4.04 a | 4.79±0.05a | 46.04±2.00b | 37.40±2.91 b | 42.76±3.31 b | 0.84±0.03 a | 0.58±0.02 a | 19.74±0.37 a | 26.11±0.80 a | 315.08±59.64 a | 112.77±19.01 ab |
|  | S6-1 | 12.18±0.83 a | 4.88±0.15a | 44.48±1.74b | 41.65±1.67 ab | 53.34±4.02 b | 0.80±0.06 a | 0.58±0.02 a | 19.87±0.18 a | 24.24±0.97 a | 76.57±0.63 b | 66.17±1.40 c |
|  | UW4 | 16.22±2.31 a | 4.71±0.05a | 45.83±3.16b | 40.64±0.54 ab | 53.18±2.00 b | 0.85±0.01 a | 0.59±0.01 a | 19.76±0.23 a | 25.98±0.26 a | 335.06±1.43 a | 133.97±9.12 a |
| R | CK | 16.81±1.53 a | 5.03±0.08c* | 53.42±0.67a | 27.42±0.48 b* | 70.55±2.45 b* | 0.84±0.03 ab | 0.62±0.03 c | 20.39±0.17 a | 27.66±1.39 b | 273.40±26.99 a | 106.94±12.75 a |
|  | NC | 14.45±4.04 a | 5.20±0.04b* | 50.38±1.49a | 29.99±3.49 ab | 67.76±3.61 b* | 0.87±0.02 a | 0.69±0.01 ab* | 20.38±0.34 a | 32.02±1.43 a* | 344.32±73.07 a | 113.54±6.49 a |
|  | S6-1 | 12.18±0.83 a | 5.79±0.03a* | 53.07±1.26a* | 35.82±2.49 a | 81.74±2.89 a* | 0.77±0.02 b | 0.65±0.01 bc* | 19.42±0.23 a | 27.44±0.31 b* | 118.99±7.19 b* | 67.26±0.88 b |
|  | UW4 | 16.22±2.31 a | 5.63±0.04a* | 49.14±2.16a | 30.42±1.51 ab* | 72.11±1.06 b* | 0.86±0.01 a | 0.74±0.03 a* | 19.39±0.52 a | 32.21±0.52 a* | 365.39±22.72 a | 123.05±18.60 a |

SM: soil moisture; TN: total nitrogen; TP: total phosphorus; TK: total potassium; AN: available nitrogen; AP: available phosphorus; AK: available potassium. Different letters indicate statistically significant differences (p<0.05) in the same row by Duncan test. *Statistically significant difference (p<0.05) for the B and R soil samples under the same inoculation conditions by T-test.

Table S3 Summary of the Miseq sequencing data and taxa identification

| Samples | Number of high-quality reads | Mean number of high-quality reads | Number of genera | Number of families | Number of  orders | Number of classes | Number of  phyla | Coverage  (%) | Number of OTUs |
| --- | --- | --- | --- | --- | --- | --- | --- | --- | --- |
| BCK_1 | 43962 | 39929±2736b | 291 | 175 | 110 | 49 | 21 | 99.67±0.03a | 870±24ab |
| BCK_2 | 34710 |  | 276 | 166 | 110 | 48 | 20 |  |  |
| BCK_3 | 41115 |  | 277 | 173 | 110 | 44 | 20 |  |  |
| BNC_1 | 28865 | 29056±908c | 263 | 160 | 104 | 43 | 19 | 99.47±0.04b | 831±34ab |
| BNC_2 | 27588 |  | 237 | 143 | 93 | 41 | 17 |  |  |
| BNC_3 | 30715 |  | 303 | 174 | 114 | 49 | 20 |  |  |
| BS6_1 | 46122 | 45941±203a | 347 | 206 | 123 | 53 | 22 | 99.65±0.06a | 948±57a |
| BS6_2 | 45537 |  | 308 | 187 | 116 | 51 | 22 |  |  |
| BS6_3 | 46165 |  | 279 | 164 | 103 | 45 | 18 |  |  |
| BUW_1 | 33075 | 32849±2286c | 270 | 163 | 108 | 45 | 20 | 99.55±0.05ab | 799±16b |
| BUW_2 | 36691 |  | 253 | 149 | 91 | 40 | 19 |  |  |
| BUW_3 | 28781 |  | 245 | 150 | 97 | 41 | 16 |  |  |
| RCK_1 | 45363 | 45185±1358b | 316 | 192 | 115 | 47 | 20 | 99.67±0.06a | 911±50a |
| RCK_2 | 42749 |  | 277 | 173 | 108 | 45 | 20 |  |  |
| RCK_3 | 47442 |  | 285 | 174 | 108 | 47 | 20 |  |  |
| RNC_1 | 53916 | 53476±373a* | 267 | 155 | 100 | 42 | 19 | 99.84±0.09a* | 710±50a |
| RNC_2 | 53776 |  | 286 | 176 | 113 | 53 | 22 |  |  |
| RNC_3 | 52735 |  | 234 | 143 | 92 | 39 | 17 |  |  |
| RS6_1 | 54119 | 57402±1846a* | 280 | 172 | 108 | 45 | 20 | 99.70±0.06a | 925±54a |
| RS6_2 | 60505 |  | 277 | 165 | 111 | 50 | 22 |  |  |
| RS6_3 | 57581 |  | 323 | 196 | 125 | 53 | 22 |  |  |
| RUW_1 | 54574 | 54682±82a* | 232 | 139 | 89 | 36 | 16 | 99.70±0.08a | 820±91a |
| RUW_2 | 54629 |  | 291 | 176 | 110 | 48 | 22 |  |  |
| RUW_3 | 54842 |  | 315 | 185 | 114 | 48 | 22 |  |  |

Different letters indicate statistically significant differences (p<0.05) in the same row by Duncan test. *Statistically significant difference (p<0.05) for the B and R soil samples under the same inoculation conditions by T-test.

Table S4 Topological properties of co-occurrence networks among different inoculation treatments

| Treatments | Whole network | | | | | | | | Random network | | |
| --- | --- | --- | --- | --- | --- | --- | --- | --- | --- | --- | --- |
|  | Number of node | Number of edges | Modularity | Average clustering coefficient | Average path length | Network diameter | Graph density | Average degree | Modularity | Average clustering coefficient | Average path length |
| CK | 234 | 602 | 0.858 | 0.922 | 1.713 | 4 | 0.022 | 5.145 | 0.370±0.001 | 0.022±0 | 1.496±0.002 |
| NC | 183 | 217 | 0.937 | 0.861 | 1.716 | 6 | 0.013 | 2.372 | 0.606±0.001 | 0.013±0 | 1.61±0.013 |
| S6-1 | 238 | 2912 | 0.741 | 0.803 | 6.274 | 14 | 0.103 | 24.471 | 0.139±0 | 0.103±0 | 1.969±0 |
| UW4 | 211 | 2504 | 0.542 | 0.734 | 4.584 | 14 | 0.113 | 23.735 | 0.141±0 | 0.113±0 | 1.948±0 |


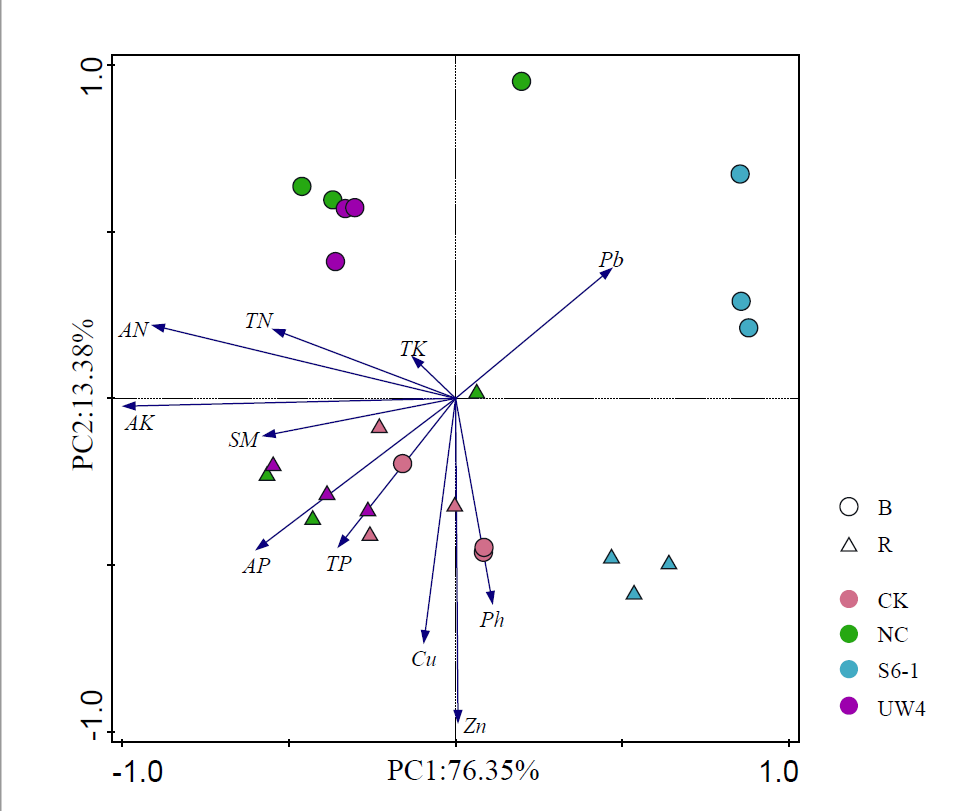


Fig. S1 Principle component analysis (PCA) of bulk (B) and rhizosphere (R) soil samples under different PGPB inoculation treatments

Fig. S2 Rarefaction curves of the observed OTUs for bulk (B) and rhizosphere (R) soil samples under different PGPB inoculation treatment
